# Associations between occupational balance, subjective health, and well-being of informal caregivers of older persons based on a cross-sectional study

**DOI:** 10.1186/s12877-022-03124-1

**Published:** 2022-05-21

**Authors:** Anna Röschel, Christina Wagner, Mona Dür

**Affiliations:** 1 Department of Health Sciences, IMC University of Applied Sciences Krems, Krems, Austria; 2 Duervation, Krems, Austria

## Abstract

**Objectives:**

Population ageing leads to a noticeable increase in demand for informal care. Informal caregivers experience high caregiver burden, such as restricted subjective health and well-being. Occupational balance is associated with subjective health and well-being. However, associations between occupational balance and subjective health and well-being of informal caregivers of older persons have not been investigated yet. Thus, the objective of this study was to explore associations between occupational balance and subjective health and well-being of informal caregivers of older persons.

**Methods:**

From September 2016 to July 2020, a cross-sectional multicenter study design was employed in Austria. Informal caregivers’ occupational balance, subjective health, and well-being as well as comorbidity of persons to be cared for were assessed with seven self-reported questionnaires. Spearman’s rank correlation coefficients *r*_s_ were calculated to determine associations between occupational balance and subjective health and well-being of informal caregivers of older persons.

**Results:**

In total 118 informal caregivers, 102 (86%) female, and their persons to be cared for, 70 (59%) female, were considered for analyses. Median age was 58 years for informal caregivers and 81 years for persons to be cared for. Informal caregivers reported restrictions in occupational balance, subjective health, and well-being. Persons to be cared for showed comorbid health conditions. Significant associations between occupational balance and determinants of subjective health and well-being were identified (*r*_s_ − 0.30 – 0.69; *p* ≤ 0.01).

**Conclusions:**

As population ageing and the demand for informal care progress, efforts to support informal caregivers and to strengthen their occupational balance, subjective health and well-being are vital.

## Introduction

The life expectancy of people is increasing worldwide. In the beginning of 2021, the number of older persons, defined as aged ≥ 60 years, was slightly over 1 billion people (13.5% of the global total population). By 2050, that number is predicted to double and to reach approximately 2 billion people [1]. At the same time, medical progress is constantly advancing, and fertility rates are falling, which additionally accelerate population ageing [2, 3]. Population ageing leads to a noticeable increase in demand for institutional health care services and informal care, whereby older persons often prefer staying in their own homes and receiving support and informal care of relatives or friends [4–9]. Informal care is defined as the provision of unpaid care (ie. feeding) by family members, relatives, or friends [10, 11].

Informal caregivers are exposed to physical and mental burden, financial pressure, and limited time resources, which lead to high caregiver burden and restricted subjective health and well-being [9, 12–16]. A construct which was found to be associated with determinants of subjective health and well-being is occupational balance, defined as the experience of a right balance between meaningful activities [17–21]. Meaningful activities describe purposeful activities a person does, wants to or has to do, such as self-care, leisure activities, household chores and work [22]. Occupational balance was found to be meaningful for informal caregivers [20, 23–32]. For instance, parents of preterm infants with a very low birthweight and parents of children with cerebral palsy experienced restrictions in their occupational balance [20, 32]. Furthermore, associations between parental occupational balance and subjective health and well-being were identified in parents of preterm infants with a very low birthweight [33] and in parents of children with cerebral palsy [20]. Additionally, an intervention to foster engagement in meaningful activities – and thereby strengthen occupational balance – led to increased subjective health in women who provided care for their spouse [31].

Informal caregivers’ occupational balance might also be relevant for the persons to be cared for [28, 34]. For example, in a randomized control trial, improved parental occupational balance was associated with weight reduction of their adipose child [34]. Moreover, mothers of children with disabilities reported their desire to maintain occupational balance for their own well-being and the well-being of their children [28]. However, to our knowledge there are no studies that focused on occupational balance of informal caregivers of older persons. Moreover, associations between occupational balance and determinants of subjective health and well-being of informal caregivers of older persons have not been investigated so far.

Thus, the objective of this study was to explore associations between occupational balance and determinants of subjective health and well-being of informal caregivers of older persons.

## Methods

### Design

A cross-sectional multicenter study design was employed to explore associations between occupational balance and determinants of subjective health and well-being of informal caregivers of older persons. Additionally, associations between occupational balance of informal caregivers and comorbidity of the persons to be cared for were explored. The study was part of a research project on occupational balance of informal caregivers (TOPIC).

### Data collection

From September 2016 to July 2020, informal caregivers of older persons were recruited personally in participating centers (University Hospital Krems, University Hospital Sankt Pölten, University Hospital Tulln, Hospital Amstetten, Hospital Mistelbach, Hospital Wiener Neustadt, Hospital Zwettl, Rehabilitationcenter Kids Chance Bad Radkersburg, Niederösterreichisches Hilfswerk and self-help groups of Bundesverband Selbsthilfe Österreich). Therefore, informal caregivers were informed about the study orally and written and were invited to participate by the research team, therapists, nurses, and self-help group leaders of the participating centers. Eligible informal caregivers were given the study information, the set of questionnaires, and an envelope to return the completed questionnaires. Additionally, informal caregivers were recruited electronically. Therefore, information about the study and an invitation to participate in the study were shared in social media and on homepages from numerous self-help groups only, by the research team and self-help group leaders. Within personal and electronic recruitment eligible caregivers were offered both, a paper- and online-based participation. The mode of participation was based on participants’ choice. Questions related to the participation were answered by the research team and those who had invited potential participants (ie. self-help group leaders). Inclusion criteria for informal caregivers for both, personal and electronic recruitment were I) the provision of informal care for a family member, relative or friend aged ≥ 60 years old at the time of participation, II) sufficient German language skills and III) the ability to complete the set of questionnaires by themselves. Implied consent was obtained. Participants confirmed to participate voluntarily by the return of the paper survey or the completion of the electronic survey. Part of the data of the current study have been used previously for the investigation of a questionnaire [33] applied in this study. Therefore, sample size calculation followed recommendations for the exploration of measurement properties with Rasch model analyses [35]. Further details on sample size calculation can be found somewhere else [33, 36].

### Measures

A literature search was conducted to identify self-reported measures which were i) used or developed to assess determinants of subjective health and well-being in informal caregivers, ii) valid and reliable, iii) available in German, and iii) had a maximum of 50 items. Additionally, the selection of measures was based on practical aspects, such as an easy use and interpretability. Eventually, seven self-reported questionnaires were selected.

Informal caregivers filled in a paper or an electronic survey of the set of self-reported questionnaires to assess sociodemographic characteristics, such as age, sex, and caring activities, and the following variables of interest: occupational balance of informal caregivers, determinants of subjective health and well-being of informal caregivers and comorbidity of the persons to be cared for. The completion of the survey took approximately 30 minutes. All instruments were validated in German language and self-applicable. Thus, participants were able to fill in the set of questionnaires at their homes without the help of a healthcare professional.

### Occupational balance

Occupational balance of informal caregivers was assessed with the "Occupational Balance in Informal Caregivers" (OBI-Care [33]) questionnaire. Within the OBI-Care occupational balance is defined as the satisfaction with occupations in different areas, their different characteristics and effects and the adaptability of these. Three subscales of the questionnaire assess aspects of occupational areas (OBI-Care OA; satisfaction with occupations in different areas), occupational characteristics (OBI-Care OC; satisfaction with characteristics and effects of occupations) and occupational resilience (OBI-Care OR; satisfaction with the adaptability of occupations). Items for each subscale are scored on a five-choice response scale and are computed into sum scores. Sum scores ranges are 5 – 45 (OBI-Care OA), 5 – 35 (OBI-Care OC) and 5 – 30 (OBI-Care OR). Low sum scores indicate satisfaction whereas high sum scores indicate dissatisfaction with one’s occupational balance [33]. Another measure on occupational balance is available in German [37]. However, the OBI-Care is the only one which was specifically developed to assess occupational balance in informal caregivers [33, 36].

### Subjective health and well-being

Different determinants of subjective health and well-being, as defined by the World Health Organization (physical, mental and social well-being [38]) were covered by the use of five self-reported questionnaires, which are described in the following.

Physical and mental health were assessed with the 12 items version of the “Short-Form 36 Health Survey” (SF-12 [39]). Two subscales with two- to five-choice response scales assess physical health (SF-12 physical health) and mental health (SF-12 mental health). Physical and mental health are determined by limitations in physical, everyday and social activities due to physical or emotional health problems, pain, general mental health and health perception as well as vitality. Total scores ranging from 0 to 100 are calculated for each subscale, whereby high scores indicate no restrictions in physical or mental health [39, 40].

Anxiety, defined as agitation and concern, and depression, defined as euthymia and dysthymia [41], were assessed with the “State-Trait Anxiety Depression Inventory” (STADI [42]). The level of anxiety (STADI anxiety) and depression (STADI depression) currently experienced by a person is scored on a four-choice response scale, ranging from 10 to 40. High scores indicate high levels of anxiety and stress whereas low scores indicate low levels [42].

Stress was assessed with the “Recovery-Stress Questionnaires” (RESTQ [43]). The definition of stress is based on a bio-psychological model and refers to a state of dysregulation (homeostatic or allostatic) as a response to stressors or inadequate demands [44]. The level of stress is rated on a six-choice response scale. Total scores range from 0 to 6. High scores indicate a high level of stress, low scores a low level of stress [43].

Subjective burden in informal caregivers was assessed with the “Burden Scale for Family Caregivers” (BSFC [45]). Caregiver burden is defined as the perception of stress related to caregiving within the BSFC [46]. Subjective burden is scored on a four-choice response scale with sum scores ranging from 0 to 84. High scores mean higher subjective caregiver burden [45].

Social support was assessed with the “Social Support Questionnaire” (SSQ [47]). Social support is understood as an individual's appraisal of the sufficiency of her or his backing system [48]. Social support is rated on a five-choice response scale. Achievable mean scores range from 0 to 5, high scores indicating high perception of social support [47].

Selected measures were found to be valid and reliable [33, 36, 41, 44, 46, 49, 50]. Information on existing cut off points of the selected measures can be found elsewhere [42, 44, 47, 51, 52].

### Comorbidity

Comorbidity of the person to be cared for was assessed with an adapted version of the “Self-Administered Comorbidity Questionnaire – German” (SCQ-D [53]). The SCQ-D addresses 14 body systems and the occurrence of health conditions, received treatment, and its impact on functioning on a two-choice response scale. Total scores range from 0 to 45 whereby a high score indicates the presence of multiple health conditions, received treatment and a high effect on functioning [53]. The SCQ-D was found to be a valid measure previously [53, 54]. However, there was no caregiver version of this commonly used questionnaire to assess comorbidities available. Therefore, the SCQ-D was adapted for the application by caregivers (caregiver-administered instead of self-administered; eg. “does the person you care for receive treatment for XY” instead of “do you receive treatment for XY”) by one of the authors with permission of the authors of the original questionnaire.

### Data analyses

Data was entered in a “Statistical Package of Social Sciences” (SPSS [55]) data file for data analyses. Data of participants who did not fill in the OBI-Care completely was excluded for analyses. Due to a non-normal distribution of all variables, medians and interquartile ranges were calculated to describe the data and nonparametric tests were conducted for further analyses. Potential differences among female and male participants were explored with Mann-Whitney U tests for independent samples [56]. Associations between informal caregivers’ occupational balance (OBI-Care OA, OBI-Care OC, and OBI-Care OR) and physical health (SF-12 physical health), mental health (SF-12 mental health), depression (STADI depression), anxiety (STADI anxiety), stress (RESTQ), caregiver burden (BSFC), perceived social support (SSQ) and comorbidity of the person to be cared for (SCQ-D) were determined with Spearman’s rank correlation coefficients (*r*_s_). We interpreted *r*_s_ ≤ 0.30 as weak, *r*_s_ = 0.31 – 0.69 as moderate and *r*_s_ ≥ 0.70 as strong associations. The level of statistical relevant significance was set at Alpha = 0.05 [56].

### Ethical considerations

The ethics committee of Lower Austria authorized the current study (number GS1-EK-4/392-2016). Participants confirmed to participate voluntarily by the return of the paper survey or the completion of the electronic survey.

## Results

### Participants

Among two hundred seventeen informal caregivers that participated in this study, 21 participants were excluded due to missing data and further 78 participants were excluded because they gave care to persons < 60 years old. Subsequently, data of 118 informal caregivers and their persons to be cared for were considered for data analyses. Characteristics on included informal caregivers and persons to be cared for are presented in Table 1.

**Table 1:** Characteristics

Informal caregivers	Female	Male	Total
Sex n (%)	102 (86)	16 (14)	118 (100)
Age in years median (IQR)	58.0 (52.0 – 64.0)	60.5 (52.0 – 73.0)	58.0 (52.0 – 64.0)
Caring activities for more than one person n (%)	48 (47)	7 (44)	55 (47)
Caring effort^a^ n (%)			
low	20 (20)	6 (38)	26 (22)
high	82 (80)	10 (62)	92 (78)
Caring activities^b^ n (%)			
body care and hygiene	75 (74)	9 (56)	84 (71)
household activities	94 (92)	16 (100)	110 (93)
cooking	83 (81)	9 (56)	92 (78)
feeding activities	66 (65)	9 (56)	75 (64)
participation in society	76 (75)	10 (62)	86 (73)
further activities	52 (51)	10 (62)	62 (53)
Occupational balance median (IQR)			
OBI-Care OA^c^	29.0 (24.0 – 33.3)	25.0 (19.3 – 30.0)	29.0 (23.0 – 33.0)
OBI-Care OC^c^	21.0 (18.8 – 24.0)	19.0 (12.3 – 22.0)	21.0 (18.0 – 24.0)
OBI-Care OR^c^	18.0 (15.0 – 22.3)	15.5 (10.5 – 19.0)	18.0 (14.8 – 22.0)
Subjective health and well-being median (IQR)			
SF-12 physical health	47.4 (38.8 – 53.6)	50.3 (46.8 – 56.4)	48.0 (39.9 – 54.1)
SF-12 mental health	40.5 (29.8 – 53.1)	53.0 (37.9 – 55.1)	42.7 (30.9 – 53.7)
STADI depression^c^	24.0 (18.3 – 28.0)	17.0 (14.0 – 21.5)	23.0 (18.0 – 28.0)
STADI anxiety	21.0 (15.0 – 26.0)	18.0 (14.8 – 27.0)	20.5 (15.0 – 26.0)
RESTQ stress	2.6 (1.7 – 3.5)	2.4 (1.3 – 2.8)	2.4 (1.6 – 3.4)
BSFC burden^c^	43.0 (31.9 – 55.0)	37.5 (16.8 – 45.8)	42.0 (29.8 – 54.0)
SSQ social support	3.4 (2.9 – 4.0)	3.6 (3.4 – 4.8)	3.5 (3.0 – 4.1)
**Persons to be cared for**			
Sex n (%)	70 (59)	48 (41)	118 (100)
Age in years median (IQR)	85 (76.0 – 89.3)	76.5 (67.0 – 85.0)	81 (71.0 – 87.3)
SCQ-D comorbidity median (IQR)	10.0 (7.8 – 15.0)	12.0 (7.5 – 19.8)	11.0 (7.8 – 15.0)
SCQ-D health conditions^b^ n (%)			
SCQ-D heart disease	31 (44)	16 (33)	47 (40)
SCQ-D high blood pressure	31 (44)	25 (51)	56 (48)
SCQ-D lung disease	19 (27)	16 (33)	35 (30)
SCQ-D diabetes	16 (23)	19 (40)	35 (30)
SCQ-D ulcer or stomach disease	34 (49)	18 (38)	52 (44)
SCQ-D kidney disease	13 (19)	10 (21)	23 (20)
SCQ-D liver disease	4 (6)	2 (4)	6 (5)
SCQ-D anemia or other blood disease	16 (23)	9 (19)	25 (21)
SCQ-D cancer	9 (13)	8 (17)	17 (14)
SCQ-D depression	37 (53)	23 (48)	60 (51)
SCQ-D osteoarthritis, generative arthritis	22 (31)	10 (21)	32 (27)
SCQ-D backpain	33 (47)	20 (42)	53 (45)
SCQ-D rheumatoid arthritis	12 (17)	3 (6)	15 (13)
SCQ-D other medical problems	46 (66)	31 (65)	77 (65)

**Abbreviations:** ^a^ = single answer; ^b^ = multiple answers; ^c^ = significant gender differences; *BSFC* Burden Scale for Family Caregivers, *OBI-Care* Occupational Balance in Informal Caregivers Questionnaire, *OA* Occupational areas, *OC* Occupational characteristics, *OR* Occupational resilience, *RESTQ* Recovery-Stress Questionnaires, SF-12 12 Item Short Form Health Survey 36, *SD* Standard deviation, *SCQ-D* Self-Administered Comorbidity Questionnaire – German, *SSQ* Social Support Questionnaire, *STADI* State-Trait Anxiety Depression Inventory

Informal caregivers experienced restrictions in occupational balance, mental and physical health, respectively. Additionally, they reported moderate to high levels of anxiety, depression, stress and caregiver burden and some limitations in social support (Table 1.). Significant differences in occupational balance, depression and caregiver burden were identified between female and male informal caregivers. Female informal caregivers reported less satisfaction with their occupational balance and higher levels of depression and caregiver burden than male informal caregivers. Persons to be cared for had various comorbid health conditions (Table 1.).

### Occupational balance and subjective health

Significant associations between caregivers’ occupational balance and subjective health, including physical and mental health, anxiety, depression, stress, caregiver burden and social support were identified (Table 2.).

**Table 2:** Spearman’s rank correlation coefficients occupational balance and subjective health and well-being

Occupational Balance	Subjective health and well-being						
	SF-12 physical health	SF-12 mental health	STADI depression	STADI anxiety	RESTQ stress	BSFC burden	SSQ social support
OBI-Care OA	− 0.193	− 0.684^*^	0.685^*^	0.616^*^	0.638^*^	0.669^*^	− 0.556^*^
OBI-Care OC	− 0.304^*^	− 0.606^*^	0.675^*^	0.594^*^	0.592^*^	0.627^*^	− 0.493^*^
OBI-Care OR	− 0.097	− 0.599^*^	0.614^*^	0.439^*^	0.445^*^	0.546^*^	− 0.418^*^

**Abbreviations:** bold* = correlation is significant (2-tailed); *BSFC* Burden Scale for Family Caregivers, *OBI-Care* Occupational Balance in Informal Caregivers Questionnaire, *OA* Occupational areas, *OC* Occupational characteristics, *OR* Occupational resilience, *RESTQ* Recovery-Stress Questionnaires, *SF-12* 12 Item Short Form Health Survey 36, *SSQ* Social Support Questionnaire - Short form, *STADI* State-Trait Anxiety Depression Inventory

Occupational balance and physical and mental health were associated significantly. OBI-Care OC (*r*_s_ = − 0.30, *p* ≤ 0.01) were weakly associated with SF-12 physical health. This indicated that a high satisfaction with occupational characteristics were related to good physical health. No significant associations between occupational areas and occupational resilience and physical health were found. OBI-Care OA (*r*_s_ = − 0.68, *p* ≤ 0.01), OBI-Care OC (*r*_s_ = − 0.60, *p* ≤ 0.01 and OBI-Care OR (*r*_s_ = − 0.60, *p* ≤ 0.01) were moderately associated with SF-12 mental health, meaning that a high satisfaction with occupational balance was related to good mental health.

Additionally, occupational balance was significantly associated with anxiety and depression. OBI-Care OA (*r*_s_ = 0.69, *p* ≤ 0.01), OBI-Care OC (*r*_s_ = 0.68, *p* ≤ 0.01) and OBI-Care OR (*r*_s_ = 0.61, *p* ≤ 0.01) were moderately associated with STADI anxiety. OBI-Care OA (*r*_s_ = 0.61, *p* ≤ 0.01), OBI-Care OC (*r*_s_ = 0.59, *p* ≤ 0.01) and OBI-Care OR (*r*_s_ = 0.44, *p* ≤ 0.01) were moderately associated with STADI depression. This implied that a high satisfaction with occupational balance was associated with low levels of anxiety and depression.

Further significant associations were identified between occupational balance, stress, and caregiver burden. OBI-Care OA (*r*_s_ = 0.64, *p* ≤ 0.01), OBI-Care OC (*r*_s_ = 0.60, *p* ≤ 0.01) and OBI-Care OR (*r*_s_ = 0.45, *p* ≤ 0.01) were moderately associated with RESTQ stress. OBI-Care OA (*r*_s_ = 0.67, *p* ≤ 0.01), OBI-Care OC (*r*_s_ = 0.63, *p* ≤ 0.01) and OBI-Care OR (*r*_s_ = 0.55, *p* ≤ 0.01) were moderately associated with BSFC burden, indicating that a high satisfaction with occupational balance was related to a low level of stress and low caregiver burden.

Moreover, occupational balance was significantly associated with social support. OBI-Care OA (*r*_s_ = − 0.56, *p* ≤ 0.01), OBI-Care OC (*r*_s_ = − 0.49, *p* ≤ 0.01) and OBI-Care OR (*r*_s_ = − 0.42, *p* ≤ 0.01) were moderately associated with SSQ social support. This means, that a high satisfaction with occupational balance was associated with high social support.

### Occupational balance and comorbidity

There was no evidence for significant associations between caregivers’ occupational balance and comorbidity of the persons to be cared (Table 3).

**Table 3:** Spearman’s rank correlation coefficients occupational balance and comorbidity

Occupational Balance	Comorbidity
	SCQ-D comorbidity
OBI-Care OA	0.093
OBI-Care OC	− 0.007
OBI-Care OR	0.047

Abbreviations: bold* = correlation is significant (2-tailed), OBI-*Care* Occupational Balance in Informal Caregivers Questionnaire, *OA* occupational areas, *OC* occupational characteristics, *OR* occupational resilience, *SCQ-D* Self-Administered Comorbidity Questionnaire – German

## Discussion

In this study we assessed occupational balance, subjective health and well-being of informal caregivers and determined associations between these variables. To our knowledge, this is the first study that focused on occupational balance of informal caregivers of persons aged ≥ 60, independent of a specific diagnosis.

In line with other studies, we identified restrictions in caregivers’ occupational balance, subjective health, and well-being. Yet (healthy) informal caregivers are essential for the health care sector, since they provide a high proportion of care for older persons. This unpaid care is not to be underestimated as it accounts for the greatest part of total care costs [14]. In the upcoming years, population ageing and the demand for informal care will progress and even accelerate [2, 3, 14]. Therefore, early detection of caregiver burden, such as restricted subjective health and well-being, is crucial to set interventions that prevent informal caregivers from getting overburdened and in need of care themselves [14].

In previous studies, occupational balance of healthy persons [19, 57, 58], informal caregivers [20, 32] and persons with various health conditions [17, 57, 59] was found to be associated with subjective health and well-being. The demonstrated associations between informal caregivers’ occupational balance, subjective health and well-being in our study support this existing evidence. Nevertheless, there is a lack of interventions to strengthen informal caregivers’ occupational balance that might also increase subjective health and well-being [30, 31].

To our knowledge, occupational balance of informal caregivers of older persons has not been investigated so far. Also, occupational balance of older persons who provide care to others has not been explored so far. However, associations between older persons occupational balance and determinants of health have been found previously. For example, a study on time use as an indicator of occupational balance found an association between a balanced amount of time use in work and leisure activities and well-being [21]. Another study found associations between occupational balance and quality of life in male nursing home residents [60] and another one between occupational balance, subjective health, quality of health and further determinants of health in community-dwelling adults [19].

Social and welfare services do commonly address the management and organization of informal care, related services and costs. Health care services typically focus on informal caregivers’ abilities to provide care. Even though, the awareness about the importance, health care services to improve caregivers' health and well-being are scarce. Based on the importance of occupational balance and its relation to health and well-being, caregivers’ occupational balance should be targeted within health care services. Occupational therapists are experts in occupational balance and set interventions to strengthen occupational balance [24]. Along with other studies on occupational balance [24, 25, 30] we agree on the need for more interventions to strengthen informal caregivers’ occupational balance, which could be delivered from occupational therapists.

As indicated in previous studies, informal caregivers’ occupational balance might not only have an impact on their own subjective health and well-being, but also on subjective health and well-being of the persons to be cared for [28, 34]. However, contrary to a study with informal caregivers of underaged persons [34], we could not identify significant associations between informal caregivers’ occupational balance and health conditions of the persons to be cared for. It must be considered that we focused on comorbidity of persons to be cared for exclusively. Associations between informal caregivers’ occupational balance and selected health conditions of persons to be cared for need to be investigated in further studies.

### Strengths and limitations

Our study showed strengths and limitations. The multicenter design yield to a high diversity of caregivers and persons to be cared for. Over 85% of participants were female, which approximately represents the informal caregiver population in Austria [16], where data collection took place. International studies also reported that informal care is mainly provided by women [61]. The application of validated self-reported questionnaires ensured the validity of collected data. Moreover, we only collected data that were indispensable for the study objective to minimize the time required for participation. Data on caregivers’ potential diagnoses and medical treatment could have provided other important insights regarding their health and well-being and the relation to occupational balance. However, it has to be considered that informal caregivers often lack time resources [16, 62] and it thus could be that severely affected informal caregivers did not participate in this study. Another limitation of this study is that, following an explorative approach, we conducted correlation analysis exclusively and we did therefore not adjust for multiple testing. Thus, the results of this study have an explorative character as well. Further studies are required to define the direction and effect size of associations between informal caregivers’ occupational balance, subjective health and well-being [63, 64].

## Conclusion

Informal caregivers’ occupational balance was associated with the determinants of subjective health and well-being in the current study. The findings align with previous studies. Others have already highlighted the informal caregivers’ risk of having a lack of occupational balance and the need for occupational balance interventions. Therefore, we suggest that existing occupational balance interventions should be increasingly considered in the health care of informal caregivers. As population ageing and the demand for informal care progress, efforts to support informal caregivers and to strengthen their occupational balance, subjective health and well-being are vital.

## Data Availability

Original data from the current study contain identifiable person data. Since participants did not give consent on data sharing, only blinded data are available from the corresponding author upon reasonable request in accordance with the European General Data Protection Regulation and the competent ethic committees. Contact information for requests on data sharing are the following: Duervation, Spitalgasse 6, 3500 Krems, Austria, mona.duer@duervation.com

## Abbreviations

BSFC: Burden Scale for Family Caregivers
OBI-Care: Occupational Balance in Informal Caregivers Questionnaire
OA: Occupational areas
OC: Occupational characteristics
OR: Occupational resilience
RESTQ: Recovery-Stress Questionnaires
SCQ-D: Self-Administered Comorbidity Questionnaire – German
SF-12: 12 Item Short Form Health Survey 36
SSQ: Social Support Questionnaire - Short form
STADI: State-Trait Anxiety Depression Inventory
